# A method to implement the electrode-entropy differentiation for lithium batteries

**DOI:** 10.1016/j.mex.2020.101052

**Published:** 2020-08-31

**Authors:** Jun-Ho Cho, Guillaume Thenaisie, Cheol-Hui Park, Sang-Gug Lee

**Affiliations:** KAIST, 291 Daehak-ro Yuseong-Gu, Daejeon 34141, Korea

**Keywords:** Thermodynamic analysis, Lithium Ion batteries diagnosis, Nondestructive measurement

## Abstract

This work presents the method and apparatus for the reproduction of the electrode-entropy differentiation approach which provides a tool to nondesctructively determine the contribution of each electrode to the battery total entropy by performing a comparative study with another battery which shares the same composition for only one of its electrodes (semi-similar types).

The 2 cells must go through the same aging process so the proposed method can be applied as follow. Firstly, an optional pre-processing step is performed, followed by linear regression between capacity loss and entropy evolution over the full SOC range. Then, a correlation is computed for each SOC value between the entropy evolution of the two cells. The result of that correlation shows at which SOC the evolution of the two cells is similar. Postulating that half-cells of the same chemistry going under the same aging conditions age the same way, the common-mode highlighted by the correlation is the SOC-domain at which the entropy of the common half-cell is dominating.

This method allows improves prior art by removing the need for custom-made half-cells of the same battery composition. The feasibility of the electrode-entropy differentiation methods is postulated, and the method is detailed. A validation study is performed on a set of batteries and the method feasibility is confirmed by the results obtained.

This paper:

• Demonstrates the feasibility of extracting the entropy from the comparative study between the subject cell and a reference.
• Includes a validation test.
• Suggest application of the method in the field of electrodes diagnosis.

## Graphical abstract

**Figure:**
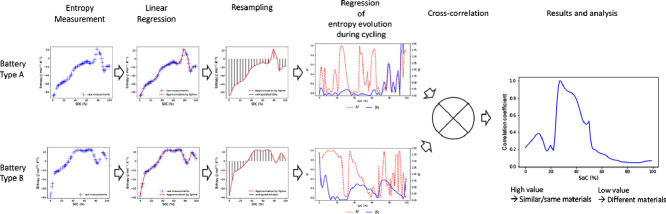

Specifications table

**Table:** 

Subject Area:	*Select one of the following subject areas:* *• Agricultural and Biological Sciences* *• Biochemistry, Genetics and Molecular Biology* *• Chemical Engineering* *• Chemistry* *• Computer Science* *• Earth and Planetary Sciences* *• Energy* *• Engineering* *• Environmental Science* *• Immunology and Microbiology* *• Materials Science* *• Mathematics* *• Medicine and Dentistry* *• Neuroscience* *• Pharmacology, Toxicology and Pharmaceutical Science* *• Physics and Astronomy* *• Psychology* *• Social Sciences* *• Veterinary Science and Veterinary Medicine*
More specific subject area:	*Nondestructive diagnosis of battery electrode aging*
Method name:	*Electrode entropy differentiation for lithium batteries*
Name and reference of original method:	*Thermodynamic & computational analysiss of lithium batteries, “A Combined Thermodynamics and Computational Method to Assess Lithium Composition in Anode and Cathode of Lithium Ion Batteries”, Wenyu Zhang and Lianlian Jiang and Pauline Van Durmen and Somaye Saadat and Rachid Yazami, 2016*
Resource availability:	https://www.scipy.org/*(all the toolbox is from there)*

## Rational

The thermodynamic analysis of lithium batteries is an emerging field that aims to provide a novel set of non-destructive tools to designers and engineers. Recent progress has open horizons of possibilities in terms of battery diagnosis, as the entropy profile evolution of the cell has shown a strong correlation with the battery aging mechanism [9]. Moreover, the simplicity of the measurements required tends to promise to the thermodynamic method a future both in the laboratory and on the field as a real-time diagnosis tool. But the incapacity of determining each electrode contribution to the cell total entropy evolution limits the analysis capacities of the thermodynamic approach. Before going any further, few terms must be defined for the sake of simplicity.

1. In this work, two cells are “similar” if they share the same composition, even if their sizes and shapes differ.
2. Two cells sharing the same composition for one of their electrodes and a different composition for the other electrode are categorized as “semi-similar”.
3. Aging conditions defined intensively and proportionally to the cell parameters are called “similar aging”, i.e. if two cells of capacity *C*_1_ and *C*_2_ are cycled at the same temperature with a current of *C*_1_/2 and *C*_2_/2 respectively, the aging conditions are similar. However, if they are cycled at the different temperatures, or if the cycling current is defined as an absolute value in Amperes, then the aging conditions are not similar.

[7] reported that a battery entropy is composed of the weighted sum of each electrode entropy. Two similar cells under similar aging conditions are expected to display similar evolutions of their respective entropy-profiles. Furthermore, under similar aging, two semi-similar cells are expected to show a similar evolution of their entropy profile at their common electrode. In other words, by comparing the evolutions of the entropy profiles of two semi-similar cells after both of them have been exposed to similar aging, the “common mode signal” of the profiles can be identified, which can be attributed to the common composition electrode. Thus, the contribution of each electrode to the cell total entropy evolution can be determined, and each electrode aging process can be monitored, in a non-destructive way. The possibility to determine each electrode contribution to the total entropy could prove useful in cell design for the analysis of new material or structures merits. The designers could track each electrode material evolution over time and determine the trigger of specific degradations.

## Implementation

### Entropy measurement

To compare the entropy profiles evolution of two semi-similar batteries, the batteries entropy profiles must be measured periodically during an aging process. The proposed method is using the measured entropy profiles but does not bring any novelty in the extraction itself. However, for the sake of repeatability, the entropy extraction process is detailed hereafter.

The entropy is extracted following the potentiometric method with relaxation correction described in [4], [5]. The linear regressions required by the potentiometric method are performed by the mean of the Python function linregress from the sub-library stats of the reference scipy library [10]. *It noteworthy to mention that any method to extract entropy, such as an impedance-based approach* *[11]**, is suitable for this method, provided its implementation is precise enough to detect entropy evolution over aging.*

The potentiometric setup used in this work is as follow: The cells are firstly charged to 4.2 V at 25^∘^C with an end of charge current of 50 mA. Then, after 1 hour of relaxation, the potentiometric extraction is started. As described in [4], the potentiometric method consists of a set of successive SOC changes followed by relaxation and (in this case) 2 temperature steps. The process is repeated until the battery terminal voltage after the second relaxation step reaches 2.9 V. According to the datasheet of the cells, the LiB cells could be discharged until 2.75 V and the LiPo to 2.8 V. However, to mitigate the risk of over-discharge due to the equipment error, the end of discharge voltage has been set slightly higher than the limit. Table 1 shows the details of each step of the potentiometric method.

**Table 1:** Potentiometric setup.

Step	Setting	Unit
Discharge step size	2.5	% of SOC
Discharge current	0.5	A
Relaxation time post-discharge	90	minutes
Relaxation time post temperature change	50	minutes
Number of temperature steps	2	
Temperature 1	10	Celsius degree
Temperature 1	25	Celsius degree

**Fig. 1:**
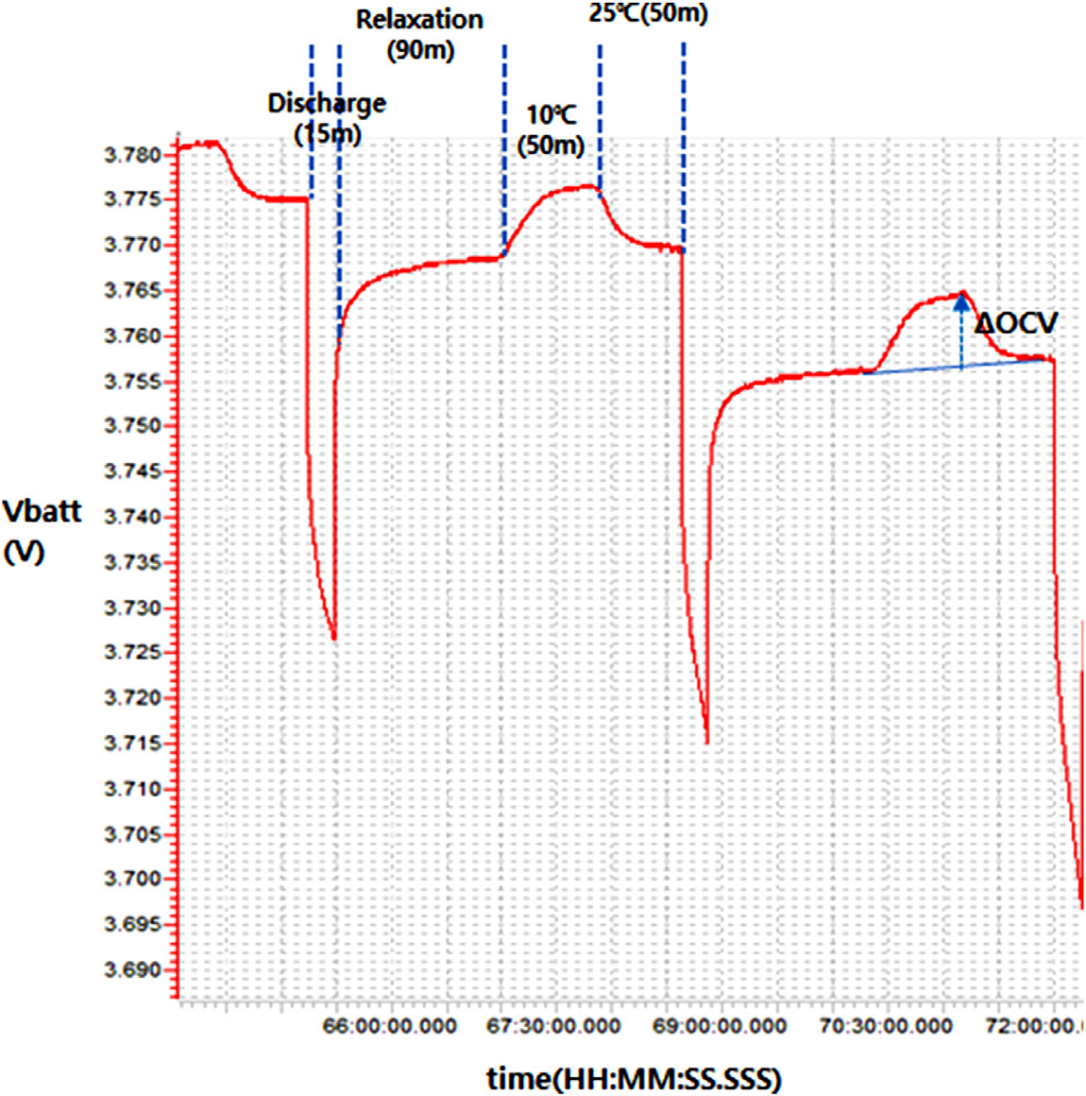
illustration of two extraction steps by potentiometric method.

Fig. 1 shows two entropy extraction steps to illustrate the extraction process.

### Pre-processing of raw data

The raw measurement data is made of tuples composed of battery temperature, voltage, and current and of a timestamp. From the laboratory measurements, at 25^∘^C, the relaxation post-discharge of a battery is a process that spreads between 40 and 60 min and which can be approximated by an exponential. The change of temperature associated with the potentiometric process takes about 40 min in a the Jeio-tech Il-11 (air medium) for the cell temperatures to be changed by 15^∘^C, following here also a pseudo-exponential. Therefore, the minimum sampling frequency (Nyquist criterion) should be higher than 1mHz for the measurement to entirely contain the full discharge relaxation and thermal relaxation processes. The battery cycler used in this experiment is suffering from limited precision and the signal is noised by environmental noises. However, as described in [1], measurement accuracy can be improved by oversampling a signal, then by applying various processing operations (filtering, decimating, …). As described in the Nyquist sampling theorem, the precision (in bits) of an ADC output can be increased by n if the sampling frequency is following the relationship:

$$f_{sampling}=4^{n}\cdot f_{Nyquist}$$

**Fig. 2:**
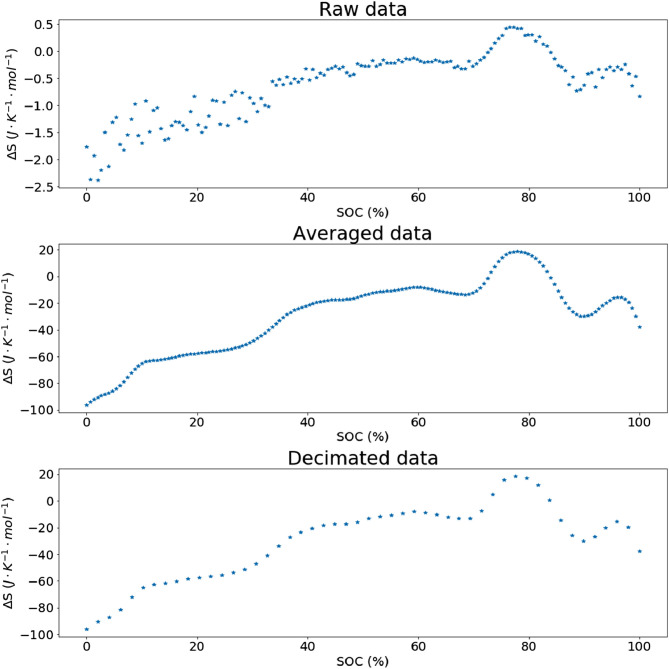
illustration of the optional preprocessing steps.

Therefore, the oversampling offers a theoretical improve of 6.6 bits of precision, which leads to a theoretical improvement by a factor 64 over the basic resolution of the ADC of the equipment. However, this improvement of resolution can be followed by a median filter (moving average) to perform an anti-aliasing operation, as described in [2], [3] which allows improving the accuracy of the measure by removing high-frequency components, which are in this situation mostly due to environmental noise. Then, to both complete the filtering process and reduce the amount of data to process, the signal is decimated by a factor 10, to a 1 Hz signal (as described in [1]). Fig. 2 illustrates the data processing steps.

It is important to note that this signal processing step is not necessary for the proposed method, and is used here to compensate for a precision-limited acquisition channel. In the case of higher precision equipment and a more stable temperature chamber, this oversampling step can be ignored.

### Core method

**Fig. 3:**
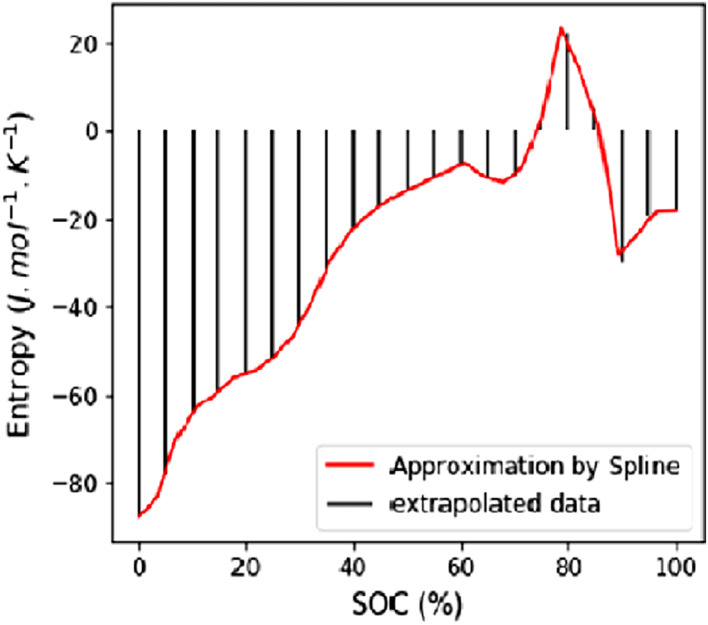
Example of a resampled dataset from measured entropy profile.

The entropy profile data of each cell at each aging step must go through a regression, such that the values of the profiles at each percent of the state of charge (SOC) can be extrapolated. To do so, a B-spline of degree 3 [14] is used as it is significantly less computational-intensive than a high-order polynomial [12] regression while offering the same level of fidelity to the measurements. The regression is performed by using the Python functions splrep and splev of the sub-library interpolate of the reference scipy library [10]. The degree of the spline has been found to be the optimum as a 1-degree spline has been found not precise enough and 5-degree spline did not show higher fidelity while requiring more computations [13], [14]. This process is called “resampling” in the rest of this work and is illustrated in Fig. 3.

Then the entropy evolution can be computed as the difference at each value of SOC between the entropy measured after n steps of cycling and the entropy of the fresh battery, as defined by

(1) $$\Delta S_{evol,n}\left(SOC\right)=\Delta S_{fresh}\left(SOC\right)-\Delta S_{n}\left(SOC\right)$$

where Δ*S*_*evol, n*_(*SOC*) is the entropy evolution value after the n-th cycle, Δ*S_fresh_*(*SOC*) the value of the fresh entropy, and Δ*S_n_*(*SOC*) the entropy measured after the nth cycle, at the given SOC.

By creating a 3-dimensions table, the entropy evolution of each cell can be expressed as a function of both SOC and cycling. Then for two semi-similar cells, at a given aging value, the cross-correlation (*ρ*) between their respective entropy evolution can be determined to highlight their common-mode signal. The entropy evolutions of cells A and B at the SOC value n at given aging and *ρ* is the correlation defined by

(2) $$\rho \left(\Delta S_{evol,A},\Delta S_{evol,B}\right)\left(SOC\right)=\sum_{m=SOC}^{100-SOC}\Delta S_{evol,A}\left(m\right)\cdot \Delta S_{evol,B}\left(SOC+m\right)$$

Because the entropy of a cell is defined by

(3) $$\Delta S_{cell}=\alpha \Delta S_{anode}+\beta \Delta S_{vathode}$$

where at a given SOC, Δ*S_cell_* is the total entropy of the cell, Δ*S_anode_* the entropy of the anode, Δ*S_cathode_* the entropy of the cathode, and *α* and *β* two numerical constants.

The evolution of the cell entropy is therefore given by:

(4) $$\Delta S_{evol,cell}=\alpha \Delta S_{evol,anode}+\beta \Delta S_{evol,cathode}$$

where Δ*S*_*evol, cell*_ is the total entropy evolution of the cell, Δ*S*_*evol, anode*_ the entropy evolution at the anode side, and Δ*S*_*evol, cathode*_ the entropy evolution at the cathode side.

Thus, the supporting hypothesis of the present work is that subject to similar aging, two semi-similar cells in which common electrode is the anode will display similar aging on their anode. Or, in other words

(5) $$\Delta S_{\mathrm{evol},A,\mathrm{anode}}≃\Delta S_{\mathrm{evol},B,\mathrm{anode}}$$

As detailed in [6], the Pearson correlation coefficient between a series X and a series Y is defined by

(6) $$r\left(X,Y\right)=\frac{n\cdot \sum \mathrm{xy}-\sum x\cdot \sum y}{\sqrt{\left(n\cdot \sum x^{2}-{\left(\sum x\right)}^{2}\right)\cdot \left(n\cdot \sum y^{2}-{\left(\sum y\right)}^{2}\right)}}$$

where n is the length of X and Y series, x is the i th element of X and y the i th element of Y. from Eq. (6) it can be seen that the correlation is symmetrical, any invariance proven on X is also proven on Y.

Assuming that X is multiplied by a scalar value *α*, a new vector Xi in which elements xi are defined by xi=αx. From Eq. (6), the correlation between Xi and Y is given by

(7) $$r\left(\mathrm{Xi},Y\right)=\frac{n\cdot \sum \mathrm{xiy}-\sum \mathrm{xi}\cdot \sum y}{\sqrt{\left(n\cdot \sum xi^{2}-{\left(\sum xi\right)}^{2}\right)\cdot \left(n\cdot \sum y^{2}-{\left(\sum y\right)}^{2}\right)}}$$

by using the expression of xi, (7) can be rewritten as

(8) $$r\left(\mathrm{Xi},Y\right)=\frac{n\cdot \sum \alpha \mathrm{xiy}-\sum \alpha \mathrm{xi}\cdot \sum y}{\sqrt{\left(n\cdot \sum \alpha xi^{2}-{\left(\sum \alpha x\right)}^{2}\right)\cdot \left(n\cdot \sum y^{2}-{\left(\sum y\right)}^{2}\right)}}$$

which can be factorized as

(9) $$\begin{array}{c} r\left(\mathrm{Xi},Y\right)=\frac{\alpha \left(n\cdot \sum \mathrm{xiy}-\sum \mathrm{xi}\cdot \sum y\right)}{\sqrt{\alpha^{2}}\sqrt{\left(n\cdot \sum xi^{2}-{\left(\sum x\right)}^{2}\right)\cdot \left(n\cdot \sum y^{2}-{\left(\sum y\right)}^{2}\right)}}\\ r\left(\mathrm{Xi},Y\right)=r\left(X,Y\right) \end{array}$$

Therefore, the value of the coefficients *α* and *β* are not impacting the correlation coefficient value as to whether these coefficients have the same value or not will not change the detection of the “common mode” of the signal entropies. And thus, the proposed method would extract the common aging feature from the aged entropy signal regardless of the specificities of the selected batteries.

Which leads to higher *ρ_norm_*(*A, B*) values at the SOC points where the anode entropy evolution is dominant over the total cell entropy evolution. By convention, a high value of normalized cross-correlation is usually defined as a value greater than 0.65 to 0.8 depending on the randomness of the studied signal. In other words, the SOC values where the cross-correlation is high are the value where the entropy evolutions of the two cells are similar, and thus the SOC values at which the common electrodes are dominating the entropy evolution. This leads to an identification of the contribution of the common-type electrode over the cell total entropy evolution, and thus by a capacity to associate an evolution of entropy to a specific electrode. Coupled with other nondestructive measurements or with a database of specific entropy evolution related to aging mechanisms, this method should grant a quick and non-destructive analysis of a cell.

## Validation

### Cells used

To assess the feasibility of the method, 2 types of batteries were tested. Each cell details are shown in Table 2.

**Table 2:** General information of the selected cells.

battery	Capacity (mA.h)	Nominal Voltage (V)	Maximal Voltage(V)	Minimum Voltage (V)	Maximum Current (A)	Manufacturer	Part Number	Package
A	700	3.7	4.2	2.75	0.7	YJ Power	ICR17335	cylinder
B	700	3.7	4.2	2.8	0.7	Nexcell	E503048	pouch

### Cycling and entropy extraction setups

• The battery data was acquired employing a battery tester (ARBIN LBT 21,084) coupled with a temperature chamber (Jeio Tech IL-11). The sampling time was of 50 milliseconds before filtering and down-sampling and of 1 second after. The voltage resolution was set at 0.01 mV, the current resolution of 0.1 mA and the temperature resolution of 0.25^∘^C.
• The cycling method selected was a series of 100 full cycles of charge and discharge by the mean of CC—CV method between 3 V and 4.2 V at 0.75C with an end-of-charge current of 50 mA, at a controlled temperature of 45^∘^C.
• The entropy was measured by the potentiometric method as described in [4] following the setup detailed in Section Implementation of this article.

### Material analysis

**Fig. 4:**
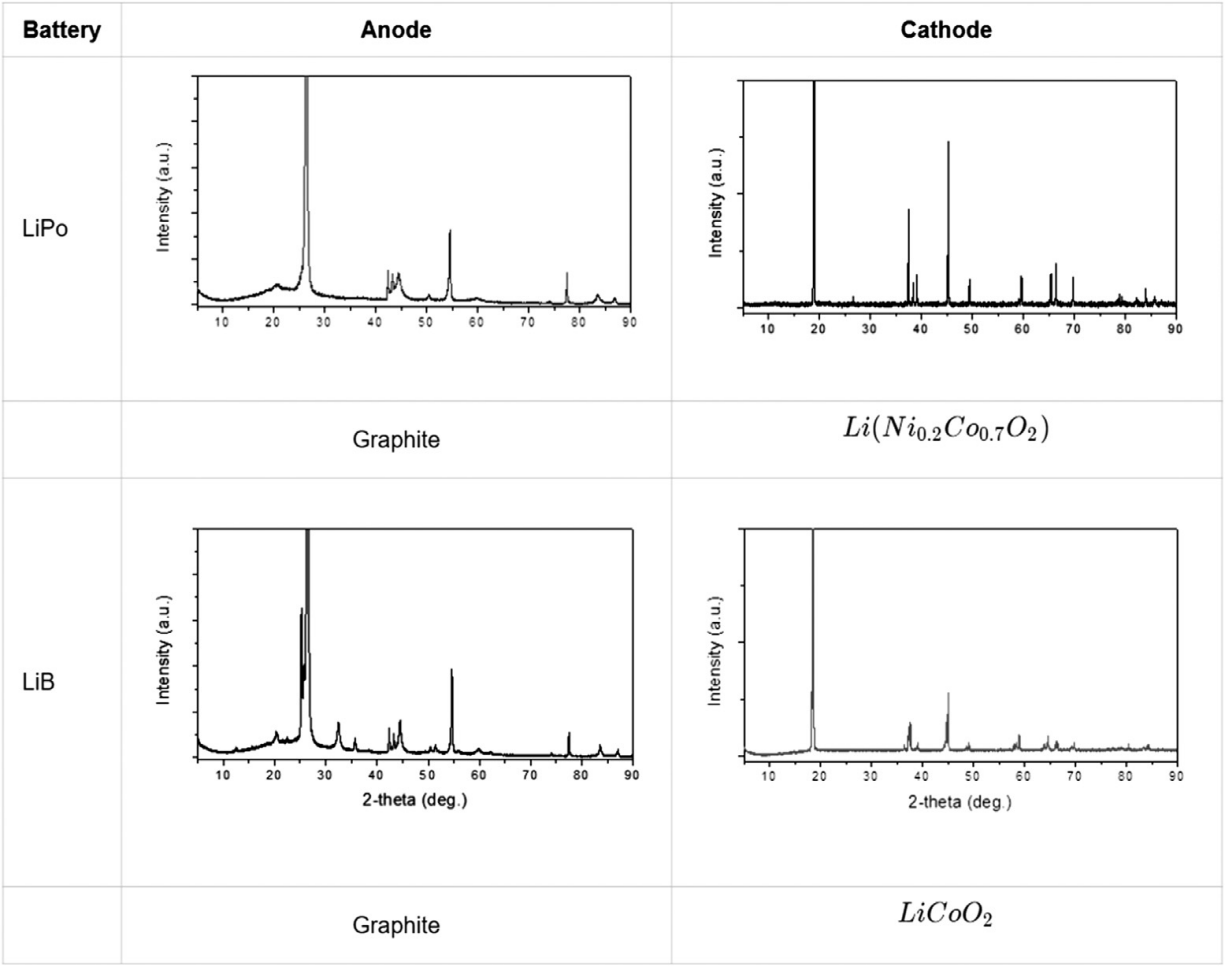
Result of the XRD analysis.

The compositions of the selected cell types were characterized by using an X-ray diffractometer (XRD) (SmartLab, RIGAKU) on a fresh unit. The batteries were discharged to 3.0 V and disassembled in an Ar-filled glove box. Subsequently, the cathode and anode electrodes were separated from the separator, washed in dimethyl carbonate (DMC), and dried. Both electrodes were scraped with the sharp blade to acquire powder state active materials. The XRD patterns were recorded in the range of 2θ=5−90 using *CuKα*1 radiation. Fig. 4 shows the results of the XRD analysis.

### Application of the method and results

**Fig. 5:**
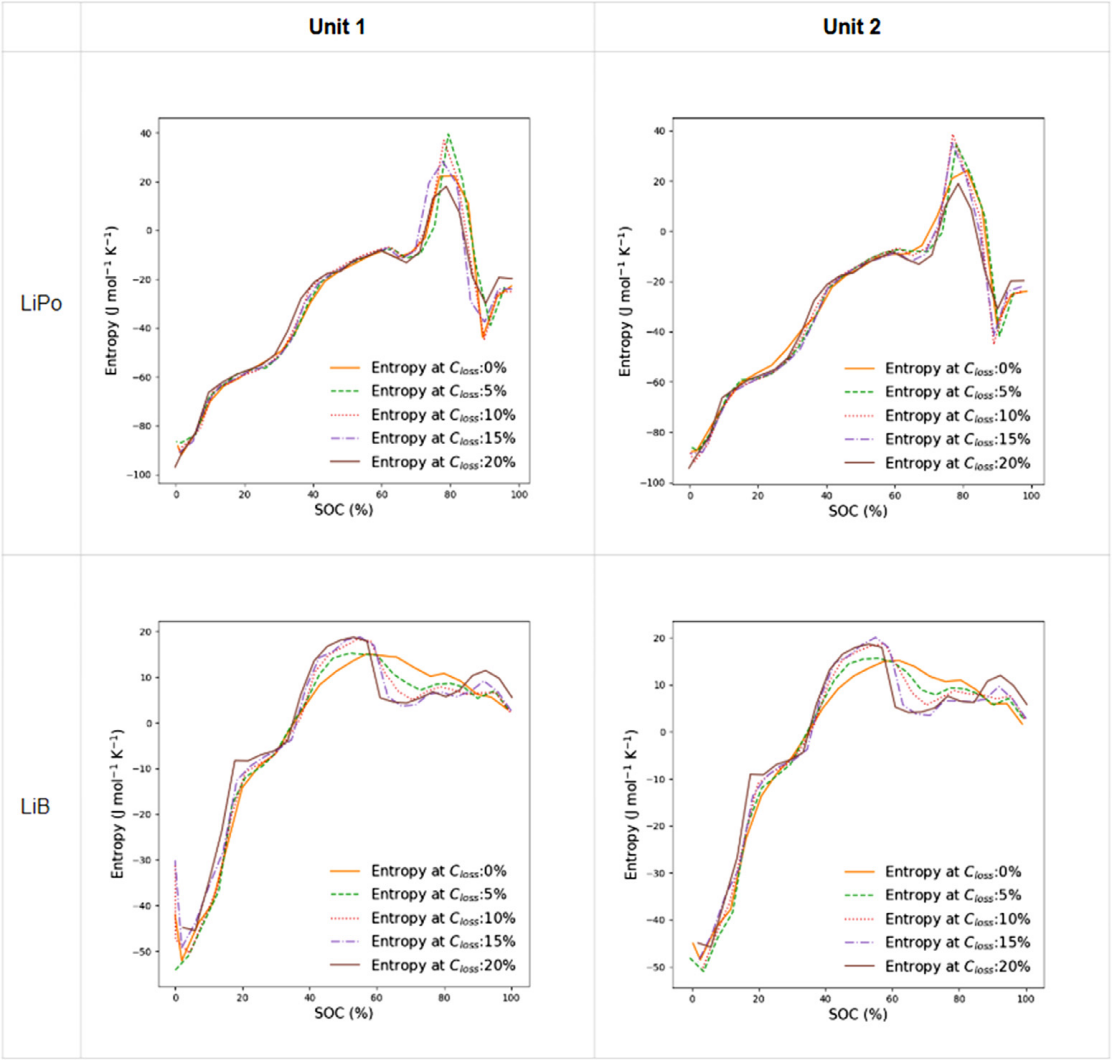
Entropy profiles of studied cells during cycling.

The measured entropy profiles were extracted every 100 cycles of aging. Then they were resampled as detailed in the previous sections. Fig. 5 shows the plots of the four cell entropy profiles plotted as a function of capacity loss over the cycling.

**Fig. 6:**
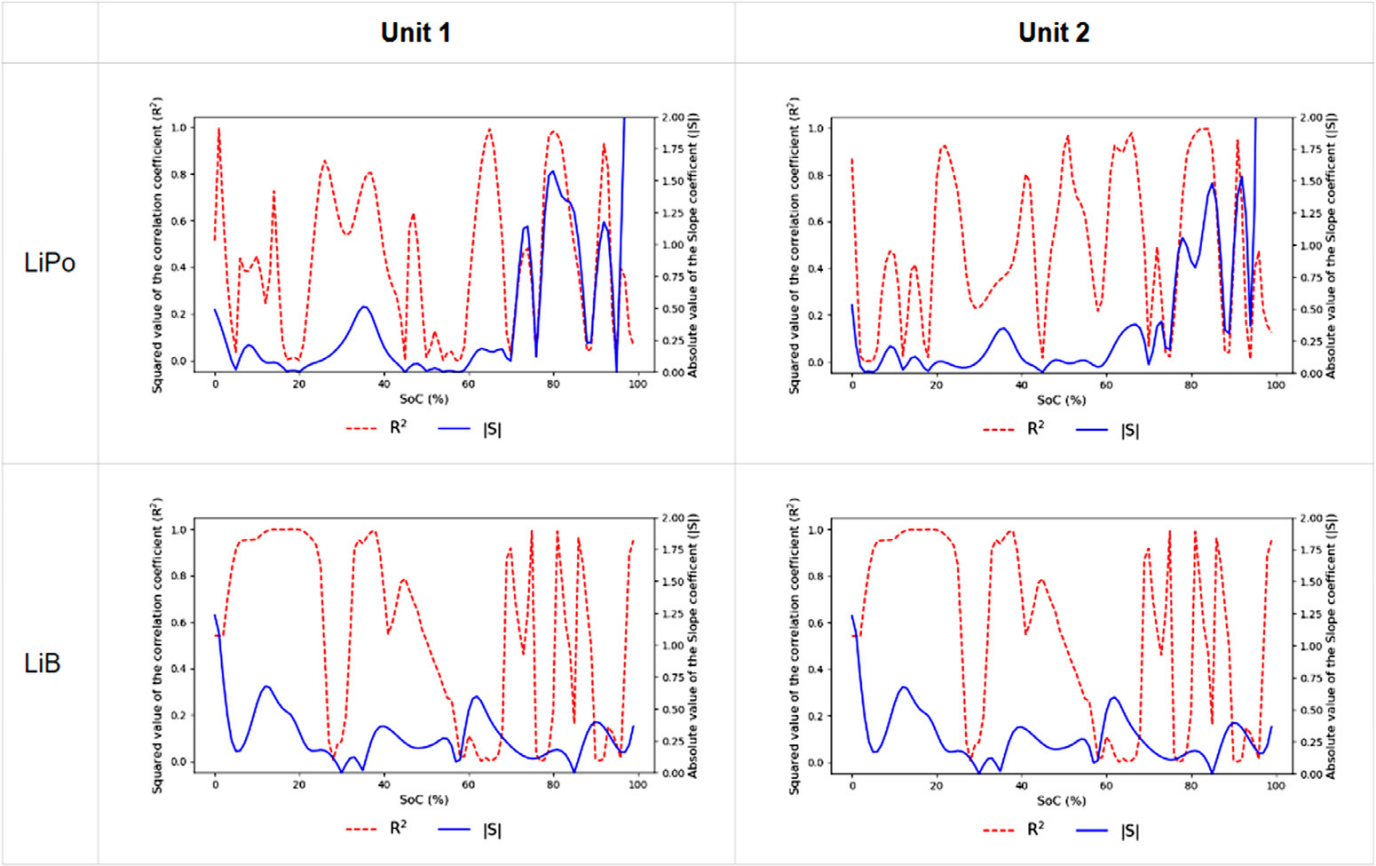
plot of the slope and correlation parameter of the linear regression as a function of SOC for each of the 4 cells.

From the resampled data, a linear regression was performed over the entropy profiles evolution as a function of capacity loss as detailed in the previous section. Fig. 6 shows the plot of the slope and correlation parameter of the linear regression as a function of SOC for each of the 4 cells.

**Fig. 7:**
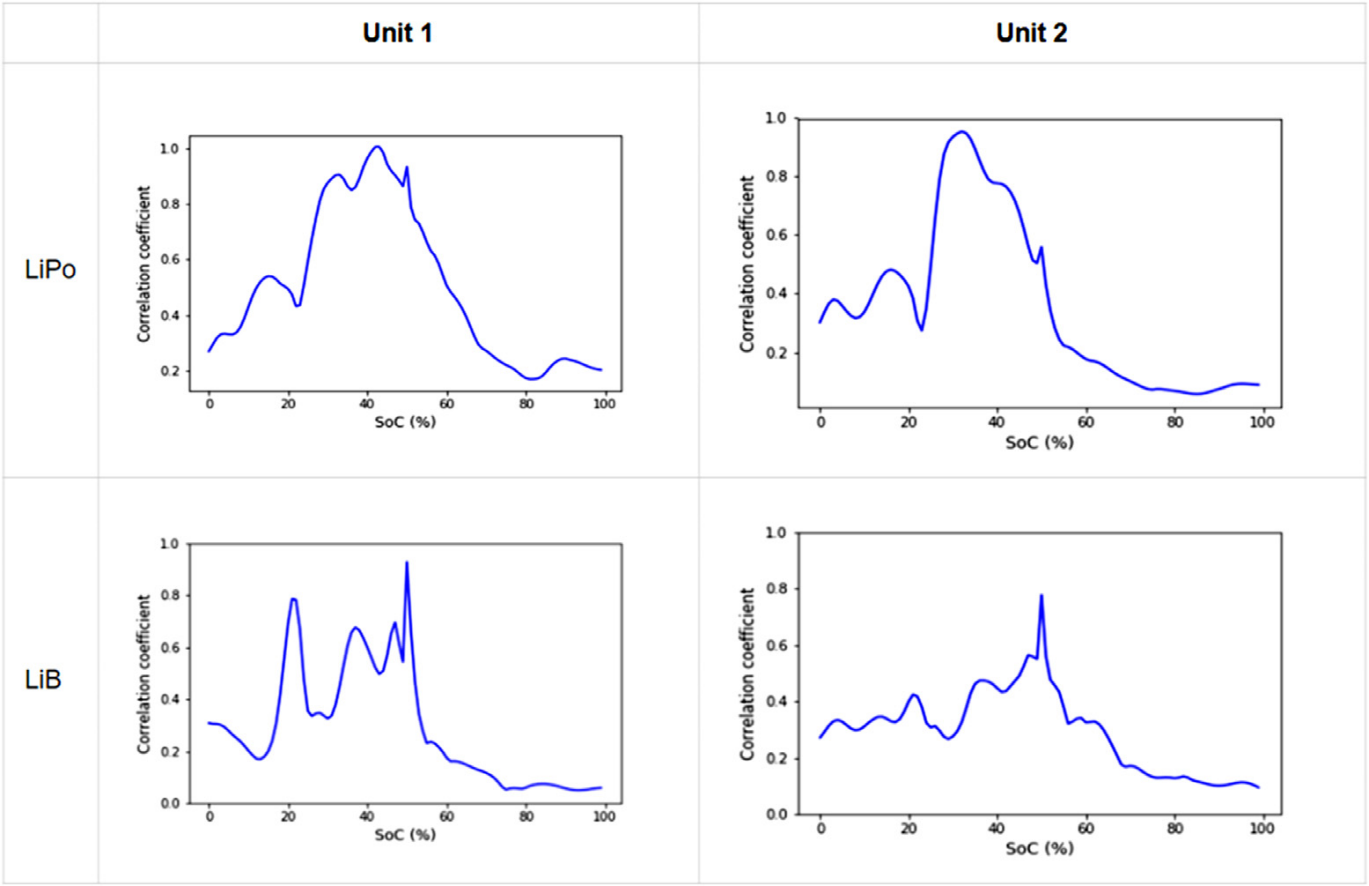
R-value as a function of SOC for the correlations between each of the studied cells.

From the aging parameters obtained, the cross-correlation is applied to the four combinations possible between 2 cells of the different types. Fig. 7. shows the correlation results obtained.

The results shown in Fig. 2 show a strong correlation at SOC values between 15 and 50%, with a sudden drop from 50 to 60% SOC. In the case of the cross-correlation between the Unit 2 of LiPo and Unit 2 of LiB, the correlation is following the same trend with a higher value at low SOC than at high SOC. However, the value is not high enough for this result to be considered as solid proof. Nevertheless, the behavior of the R-parameter of the cross-correlation shows in most of the cases that the common mode of the two cells is to be found at low SOC values. This, in agreement with the proposed hypothesis, would indicate that the entropy is dominated by the anode for SOC. Which are the results demonstrated in [7] by destructive manner. It is important to note that the proposed method is not perfect and that some source of error remains to be identified and filtered out, for the results to be 100% in agreement with the theory. On a side note, below 15% the material is too disorganized and the entropy values are too chaotic to be used reliably [8].

## Limitations

This method is limited by the fact that two semi-similar cells must be cycled under the same conditions, and thus it cannot be adopted as it is in an embedded system for failure analysis and diagnosis. Moreover, to limit the influence of variations of the battery fabrication process, multiple cells of the same type must be measured to determine the best statistical aging profile for the reference battery.

Nevertheless, this method showed promising results and has proven functional to determine the entropy contributions with good agreement to prior work. Therefore, as long as the sample of reference cells is big enough, and the cycling conditions are the same between the studied cells and their semi-similar references, the method can be applied for electrode aging analysis with the advantage of being non-destructive and relatively cheap compared to in-situ approaches.

## Availability of the data

The cycling data after preprocessing are available on-demand and will be uploaded on the server of our institution when it will be made available.

## Declaration of Competing Interest

The authors whose names are listed immediately below certify that they have NO affiliations with or involvement in any organization or entity with any financial interest (such as honoraria; educational grants; participation in speakers’ bureaus; membership, employment, consultancies, stock ownership, or other equity interest; and expert testimony or patent-licensing arrangements), or non-fi nancial interest (such as personal or professional relationships, affi liations, knowledge or beliefs) in the subject matter or materials discussed in this manuscript.

Author names:

• Guillaume Thenaisie
• Jun-Ho Cho
• Sang-Gug Lee

The authors whose names are listed immediately below report the following details of affiliation or involvement in an organization or entity with a financial or non-financial interest in the subject matter or materials discussed in this manuscript.

Author names

Cheol-Heui Park

He is currently an employee of Samsung Electronics, which is one founder of the project, can be related remotely to Samsung SDI which manufactures batteries, and has therefore general interests in the field of battery measurements.

## Appendix Supplementary materials

Image, application 1
