# Supplementary material for: A method to implement the electrode-entropy differentiation for lithium batteries
Source: MethodsX. 2020 Aug 31;7:101052. doi: 10.1016/j.mex.2020.101052 (PMC7486607; doi:10.1016/j.mex.2020.101052)
Supplement: Supplementary file 1 [file mmc1.docx]

## Supplementary material and/or Additional information

### Entropy evolution during cycling

**As a side result, which could be used in another type of study, the proposed method allows us to plot the evolution of the entropy of the cells over the cycling. The difference between fresh entropy and aged entropy is called differential entropy in this manuscript as discussed in [5]. Figure 8 shows the evolution of the differential entropy of the units 1 of the LiPo and LiB types over the aging.**


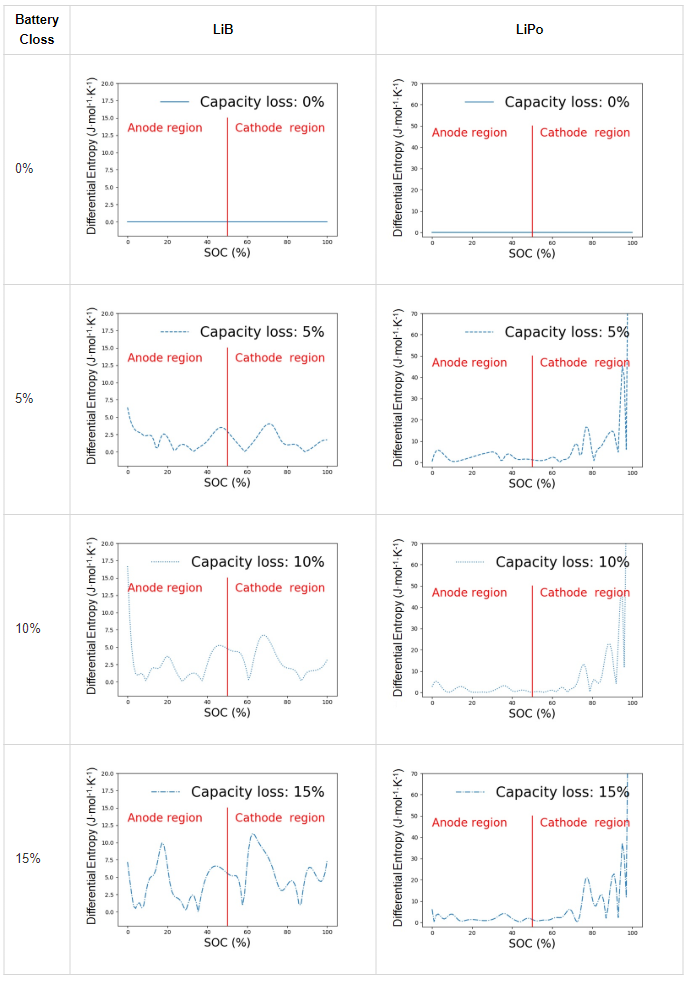


*Figure 8. Evolution of the differential entropy of the units 1 of the LiPo and LiB types over the aging*
